# The Dental Relief Fund

**Published:** 1913-12-15

**Authors:** James McManus

**Affiliations:** Hartford, Connecticut


### THE DENTAL RELIEF FUND.

BY JAMES MCMANUS, D.D.S.

After twenty years of striving to raise money for a National Relief Fund, with the kind aid of the Dental Journals that gave liberally, ample space to special appeals, Society discussions and convincing editorials advocating the movement; and no returns until the San Francisco Local Relief Committee, seven years ago, made their final report to the public, and turned back to the National Dental Association $3,969.75, the amount left after their duties ceased, and no additions from any source since but the low rate of interest. The National Relief Committe—Ex-President Dr. L. G. Noel of Nashville, Tenn., Dr. W. T. Chambers, Denver, Colo., Dr. Edward S. Gaylord, New Haven, Conn., and Dr. James McManus, Hartford, Conn., after the Kansas City meeting decided on a change of methods, and Dr. Gaylord, in consultation with Drs. James McManus, George O. McLean and B. A. Sears, Relief Committee of the Hartford Dental Society, started a personal appeal crusade.

At a meeting of the New Haven, Conn., Dental Society, October 9th, Dr. Gaylord made a statement and an appeal, and the Society voted from its Treasury a donation of $50 cash. At a meeting of the Hartford Society, October 13th, the Committee made an appeal and from thirty-two members present, they raised in cash $151. The next day, October 14th, at the annual meeting of the Northeastern Dental Society, held in Hartford, Conn., Drs. Gaylord, McManus, McLean and Sears made winning appeals, and the Society voted cash $500.00 to the fund.

The Committee are glad to tell the dentists of the country how successfully the new crusade has opened. After years of work, worry, and expense for printing and mailing circulars, with no returns—complete failure—to have in hand donations amounting to $701.00 raised by personal appeals and Dental meetings in the little State of Connecticut, gives them hope and confidence that every City and State in the Country will respond largely in advance of Connecticut, if a few of the Dentists will join the Crusaders in this special appeal campaign.

The Committee acting on the suggestion of Dr. Ottolengui, in the *Items of Interest*, July Number, 1912, will have an attractive Holiday Association Seal, designed by Dr. Charles McManus of Hartford, Conn., on sale in all the Dental Supply houses December 1st. The seal may be put on the back of letters, monthly bills, and all correspondence papers, parcel post packages, and it is the hope of the Committee that friends, managers of department and other stores, will buy liberally, and put them on a large number of the gift parcels they send out during the Holiday season. Every member of the Association is entitled to receive aid in case misfortune befalls him, if there is money enough in the Relief Fund to warrant it; and surely in the cheery Holiday season every Dentist ought to give gladly a Christmas gift to this Fund. The Association seal, happily suggested by Dr. Ottolengui, will enable every dentist in the country, at small expense, to keep in touch with one hundred dear friends, business acquaintances and happy children; all alike appreciating the cheery Christmas and New Years greetings. A liberal purchase and use of the seal will tell the public that Dentists are in line with other callings in the care of the unfortunate and aged, and the Relief Fund by January 1, 1914, will have a respectable financial standing.

Wishing all the members of the profession a Merry Christmas and many Happy New Years for the National Relief Fund Committee.

James McManus, D.D.S.,

Hartford, Connecticut.

